# Swiping Disrupts Switching: Preliminary Evidence for Reduced Cue-Based Preparation Following Short-Form Video Exposure

**DOI:** 10.3390/bs15081070

**Published:** 2025-08-06

**Authors:** Wanying Luo, Xinran Zhao, Bingshan Jiang, Qiang Fu, Juan’er Zheng

**Affiliations:** 1 China National Institute of Standardization, Beijing 100191, China; 2001110661@pku.edu.cn; 2 School of Psychological and Cognitive Sciences, Peking University, Beijing 100871, China; 2301110711@stu.pku.edu.cn (X.Z.); ivybingshan_jiang@stu.pku.edu.cn (B.J.)

**Keywords:** short-form video, cognitive flexibility, task switching, proactive control, cue-based preparation, media–cognition interaction

## Abstract

The rapid rise of short-form video platforms such as TikTok and Instagram Reels has transformed digital engagement by promoting fragmented, high-tempo swiping behaviors and intense sensory stimulation. While these platforms dominate daily use, their impact on higher-order cognition remains underexplored. This study provides preliminary behavioral experimental evidence that even brief exposure to short-form video environments may be associated with reduced cue-based task preparation, a specific subcomponent of proactive cognitive flexibility. In a randomized between-subjects design, participants (N = 72) viewed either 30 min of TikTok-style content, a neutral documentary, or no video (passive control), followed by a task-switching paradigm with manipulated cue–target intervals (CTIs). As expected, the documentary and control group exhibited significant preparation benefits at longer CTIs, reflected in reduced switching costs—consistent with effective anticipatory task-set updating. In contrast, the short video group failed to leverage extended preparation time, indicating a selective disruption of goal-driven processing. Notably, performance at short CTIs did not differ across groups, reinforcing the interpretation that reactive control remained intact, while proactive preparation was selectively impaired. These findings link habitual “swiping” to disrupted task-switching efficiency—a phenomenon summarized as swiping disrupts switching. These findings suggest that short-form video exposure may temporarily bias attentional regulation toward stimulus-driven reactivity, thereby undermining anticipatory cognitive control. Given the widespread use of short-form video platforms—especially among young adults—these results underscore the need to better understand how media design features interact with cognitive control systems.

## 1. Introduction

The proliferation of short-form video platforms such as TikTok, Instagram Reels, and Kwai has fundamentally reshaped patterns of digital media consumption. Unlike traditional long-form content, short videos are characterized by rapid scene transitions, condensed information density, and immediate sensory stimulation (Lu et al., 2022; Chen et al., 2023). This unique format encourages users to engage in rapid, continuous swiping—a behavior that promotes shallow, fast-paced processing and reactive attentional shifts (Yan et al., 2024).

While short-form video platforms have become deeply embedded in daily routines, growing concerns have emerged regarding their potential cognitive consequences, particularly their immediate impact on attentional control and executive functioning (Ophir et al., 2009). Emerging research suggests that excessive exposure to fast-paced, fragmented media environments may impair sustained attention, diminish cognitive flexibility, and promote reactive, stimulus-bound processing styles (Xu et al., 2023; David & Roberts, 2024). Although short-form video consumption is characterized by low-effort, passive information engagement, its influence may potentially be shaping how the cognitive system prepares for, and responds to, upcoming demands.

One particularly sensitive marker of such influence is cognitive flexibility, a multifaceted construct within executive functioning that enables individuals to adapt to changing task demands. This capacity involves a constellation of processes, including task-set reconfiguration, inhibition of obsolete rules, and maintenance of new goals (Miyake et al., 2000; Diamond, 2013). Within this construct, our study focuses on cue-based preparatory updating, a proactive mechanism that allows individuals to align internal task sets with external cues to optimize performance (Monchi et al., 2001; Stelzel et al., 2010).

Task-switching performance provides a well-established behavioral index of this capacity, requiring individuals to alternate between cognitive tasks governed by different rules (Monsell, 2003). Critically, task-switching efficiency depends on cue-based preparation: the ability to proactively engage task-relevant mental sets prior to target onset (Kiesel et al., 2010; Vandierendonck et al., 2010). Notably, performance depends on the ability to utilize cue–target intervals (CTIs) for anticipatory preparation: longer CTIs typically support proactive reconfiguration, reducing switching costs and enhancing flexibility (Meiran, 1996; Kiesel et al., 2010; Schmitz & Voss, 2012).

However, it remains unclear whether even brief immersion in passive, high-intensity digital environments such as short-form video platforms can disrupt these preparatory mechanisms. According to dual mechanisms theory (Braver, 2012), cognitive regulation operates through both proactive control (sustained goal maintenance) and reactive control (stimulus-driven reactivity). Environments that prioritize novelty and perceptual salience may bias cognitive control toward the latter, reducing the system’s readiness to preconfigure task sets in advance.

Although short-form video use involves frequent attentional shifts, these shifts are typically transient and externally triggered, driven by perceptual novelty rather than internal goals. This reactive attentional style may reinforce bottom-up processing, taxing working memory and diminishing the ability to sustain goal states. Neurocognitive accounts suggest that such exposure preferentially activates striatal dopaminergic circuits linked to salience and reward while downregulating prefrontal regions implicated in task-set maintenance and preparatory control (Ott & Nieder, 2019). Thus, even short-term immersion may reduce the effectiveness of anticipatory preparation—especially when task demands allow for it, as in long CTI contexts.

Given that prior work has predominantly focused on chronic media exposure, little is known about whether even brief immersion may transiently disrupt anticipatory control mechanisms. To examine this possibility, we employed a cue-based task-switching paradigm to assess whether short-form video exposure disrupts proactive preparation. Participants (N = 72) were randomly assigned to one of three groups: (1) a short video group, who viewed 30 min of TikTok-style content; (2) a documentary group, who viewed 30 min of neutral, low-tempo educational material; and (3) a no-video control group, who sat quietly for 30 min without screen-based stimuli. Following the exposure phase, participants completed a task requiring alternation between parity and magnitude judgments. Each trial featured either a short or long CTI between cue and target. Based on previous findings, we expected the documentary and no-video groups to benefit from longer CTIs through improved preparation, reflected in reduced switching costs. In contrast, we hypothesized that the short video group would show diminished or absent preparation benefits, consistent with impaired cue-based task-set updating.

This framework links habitual swiping behavior in fast-paced media contexts with the disruption of anticipatory cognitive regulation. We propose that the passive, rapid engagement style reinforced by short-form video platforms may temporarily compromise the cognitive system’s ability to reconfigure task sets and prepare for goal-directed behavior—a phenomenon metaphorically summarized as swiping disrupts switching. Task switching thus serves as a robust behavioral probe for investigating how immersive, fragmented digital media experiences affect proactive cognitive control. While task-switching performance reflects only one subcomponent of executive function, it offers a focused lens into preparatory flexibility.

By bridging habitual media behaviors with core cognitive mechanisms, this study provides preliminary evidence that short-form video exposure may selectively interfere with anticipatory task-set reconfiguration. These findings extend theoretical models of media–cognition interactions by identifying a specific pathway—proactive preparation failure—through which short video use may transiently affect cognitive performance. Given the pervasive use of such media, especially among younger populations, understanding how platform design interacts with core cognitive systems has critical implications for digital well-being, attentional health, and adaptive functioning in daily life.

## 2. Participants and Materials

### 2.1. Participants

Seventy-two healthy university students (*n* = 24 per group; short-form video group, documentary group, and no-video control group; age range: 18–22 years, M = 20.1 years, SD = 0.91; 46 females) voluntarily participated in the study. This age range was deliberately selected for both theoretical and practical considerations. First, emerging adults represent the primary demographic for short-form video platforms, with usage rates exceeding those of other age groups (Anderson et al., 2023). Second, this developmental window is characterized by peak maturation of core executive functions, including cognitive flexibility, alongside heightened neural plasticity and susceptibility to environmental modulation (Peters, 2020; Insel & Cohen, 2025). Focusing on this population thus maximizes the ecological validity and theoretical relevance of our findings.

All participants were right-handed, reported normal or corrected-to-normal vision, and had no history of neurological or psychiatric disorders. Written informed consent was obtained from all participants. The study protocol was approved by the institutional ethics committee of Peking University (Approval No. 2021-03-04) and conducted in accordance with the Declaration of Helsinki.

A priori power analysis using G*Power 3.1 (Faul et al., 2009) indicated that, under parameters of f = 0.20, α = 0.05, and power = 0.95, a sample size of 66 participants would be sufficient to detect the hypothesized Group × CTI × Trial Type interaction in a mixed-design ANOVA. Our final sample of 72 participants yields a power of 0.963, providing robust sensitivity to detect small-to-moderate interaction effects.

### 2.2. Media Stimuli

Three experimental conditions were designed to manipulate short-term digital immersion:

Short Video Condition:

Participants viewed a curated set of 120 TikTok-style videos (mean duration = 25.0 s, SD = 4.3 s) presented on a standard smartphone (6.5-inch screen, 1080p resolution) to closely simulate typical short-form video consumption. To enhance ecological validity, participants held the phone in hand and manually swiped through the videos at their own pace. The video set was constructed based on a preliminary survey involving 65 undergraduate students from Peking University. Each participant opened the TikTok app under naturalistic conditions, and the 5th to 10th recommended videos from their personalized feed were collected. This sampling strategy aimed to capture algorithmically generated, platform-representative content while minimizing idiosyncratic bias.

To ensure content appropriateness and experimental control, videos containing explicit sexual material, horror imagery, ASMR triggers, political messaging, or overt advertising were excluded. The final set consisted of clips characterized by rapid scene transitions, high visual dynamism, and emotionally engaging content—core structural features of popular short-form videos. All videos were independently reviewed to ensure compliance with ethical standards.

Documentary Condition:

Participants watched a 30 min nature documentary on the same smartphone, under identical environmental conditions. The documentary was carefully selected to contrast with short-form content along key structural parameters while matching visual resolution and audio-visual quality. Specifically, the documentary featured slower scene transitions (mean cut frequency = 1.2 cuts per 15 s), continuous narrative flow, and minimal attentional fragmentation. Pre-screening confirmed that the documentary matched the short video set in luminance and color saturation, ensuring low-level perceptual equivalence across conditions.

No-Video Control Condition:

Participants in the control group remained seated in the same environment for 30 min without any media exposure. They were instructed to sit quietly without using their phones or engaging with external stimuli. To match posture and motor engagement, participants held a rectangular object comparable in size and weight to a smartphone for the duration of the session.

All sessions were conducted individually in a quiet, well-lit laboratory room equipped with workstation cubicles separated by opaque partitions to eliminate interaction and visual contact. Media stimuli (for the short video and documentary conditions) were presented on a 27-inch monitor at a viewing distance of approximately 60 cm, with audio delivered through noise-canceling headphones to standardize auditory conditions.

### 2.3. Cognitive Task Stimuli

The cognitive flexibility task was programmed using PsychoPy software (2024.2.4) and presented on a 27-inch monitor with a refresh rate of 60 Hz at a viewing distance of approximately 60 cm.

The target stimuli consisted of centrally displayed Arabic digits (1, 2, 3, 4, 6, 7, 8, 9) presented in white against a black background. Task cues were symbolic shapes— a white triangle (“△”) indicating the parity judgment task and an inverted triangle (“▽”) indicating the magnitude judgment task—shown at the center of the screen. Participants responded using a modified keyboard with the following mapping: odd numbers on the “1” key, even numbers on the “7” key, numbers smaller than 5 on the “3” key, and numbers greater than 5 on the “9” key. All keys must be completed with the index finger of the right hand. To minimize potential semantic priming or visual cueing effects, all response keys were covered with neutral white stickers.

## 3. Design and Procedure

### 3.1. Experimental Design

The study employed a mixed factorial design with one between-subjects factor and two within-subjects factors. The between-subjects factor was Media Immersion Group (short video vs. documentary vs. no-video control). The within-subjects factors included task switching type (repeat vs. switch trials) and cue–target interval (CTI) with two levels (short: 0 ms; long: 500 ms).

Dependent variables included reaction time (RT), response accuracy (ACC; proportion of correct responses), and inverse efficiency scores (IES), which provided an integrated index of speed–accuracy trade-offs commonly used in task-switching research (Kiesel et al., 2010).

### 3.2. Procedure

All participants completed the experiment individually in a quiet, standardized laboratory environment with consistent lighting and minimal external distractions. The procedure comprised two phases: a media immersion phase followed by the cognitive task.

#### 3.2.1. Media Immersion Phase

Participants were randomly assigned to one of three groups:

Short Video Group: Viewed a curated set of TikTok-style short videos for 30 min on a handheld smartphone to replicate typical usage patterns. Participants freely swiped through the content at their natural pace.

Documentary Group: Watched a 30 min nature documentary on the same device. The documentary was selected to differ from short videos in structural characteristics (e.g., slower scene transitions, continuous narrative), while controlling for visual quality and viewing duration. Participants passively viewed the documentary without interaction.

No-Video Control Condition: Participants in the control group were seated in the same environment for 30 min without any media exposure. They were instructed to remain quietly seated, avoiding the use of phones or other stimuli. To control for motor activity and posture, participants in the no-video group held a rectangular object matched in shape and weight to a smartphone for 30 min.

Although no formal physiological or eye-tracking measures were used to quantify engagement, all sessions were conducted under direct experimenter supervision. Participants were instructed to maintain visual attention throughout, and swipe behavior in the short video group was observable to ensure active viewing.

#### 3.2.2. Cognitive Task Phase

**Figure 1:**
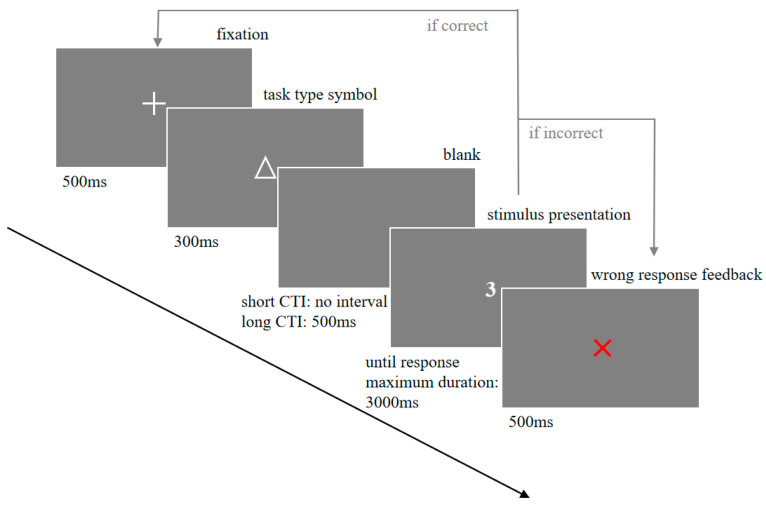
Schematic illustration of a single trial in the task-switching paradigm. Each trial began with a fixation cross presented for 500 ms, followed by a task cue (△ for parity judgment; ▽ for magnitude judgment) displayed for 300 ms. After a cue–target interval (CTI) of either 0 ms or 500 ms, an Arabic digit (1, 2, 3, 4, 6, 7, 8, or 9) appeared and remained on screen until a response or for a maximum of 3000 ms. If the response was incorrect, a red “×” was shown for 1000 ms as feedback.

Immediately following media immersion, participants completed a computerized task-switching paradigm designed to assess cognitive flexibility:

Each trial followed the sequence illustrated in Figure 1:

A central fixation cross (“+”) was displayed for 500 ms;

A symbolic task cue (“△” for parity judgment, “▽” for magnitude judgment) appeared for 300 ms;

A CTI followed (either 0 ms for the short or 500 ms for the long condition);

A digit (1, 2, 3, 4, 6, 7, 8, or 9) was presented centrally in white against a black background until a response or for a maximum of 3000 ms;

Incorrect responses triggered red “×” feedback for 500 ms.

Participants responded using a modified keyboard with concealed keys to avoid semantic priming. They were instructed to rest their finger on the central “5” key between trials to standardize motor preparation.

The cognitive task included two experimental blocks corresponding to the two CTI conditions, with block order counterbalanced across participants within and across all media groups. Each block contained 80 valid trials (50% Repeat, 50% Switch), presented in a pseudorandom sequence. Prior to the formal task, participants completed 16 practice trials to ensure task comprehension.

The entire experimental session lasted approximately 45 min.

### 3.3. Data Processing and Analysis

RTs and ACCs were recorded for each experimental condition. Data preprocessing followed established procedures in task-switching research. Specifically:

Following prior work, trials with response times below 200 ms were treated as anticipatory responses and excluded from reaction time analyses. This resulted in the removal of 34 trials in total (0.29% of all valid trials).

Error trials were excluded from RT analyses but included in accuracy computations.

IES were calculated for each condition to provide an integrated measure of speed–accuracy trade-offs, as recommended in cognitive flexibility research (Vandierendonck et al., 2010):

(1) $$IES=\frac{RT}{ACC}$$

To ensure data quality, we applied an outlier exclusion criterion based on ±3 standard deviations from the group mean IES. One participant in the short video group exceeded this threshold and was excluded from further analysis. The final sample consisted of 72 participants (*n* = 24 per group).

Prior to conducting parametric analyses, we tested the assumption of normality for each condition using the Shapiro–Wilk test on subject-level mean values of IES. All distributions were found to be not significantly different from normal (all *p*s > 0.05), indicating that the normality assumption was satisfied. Homogeneity of variance was assessed using Levene’s test, which revealed no significant violations for any of the factors (all *p*s > 0.05). These results justified the use of mixed ANOVA models in subsequent analyses.

Data analyses proceeded in two stages to comprehensively assess both global performance differences and condition-specific effects:

#### 3.3.1. Group-Level Performance Comparison

One-way ANOVA was conducted on RTs and ACCs for switch trials, collapsed across CTI conditions. This analysis evaluated whether short video immersion impaired cognitive flexibility at a general level, irrespective of preparation time.

#### 3.3.2. Condition-Specific Task-Switching Analysis

A 3 (Group: short video vs. documentary vs. no-video control) × 2 (CTI: short vs. long; within-subjects) × 2 (Trial Type: repeat vs. switch; within-subjects) mixed-design ANOVA was performed on IES, providing an integrated measure of task-switching efficiency while accounting for speed–accuracy trade-offs.

All statistical analyses were performed using SPSS (version 26.0), with significance thresholds set at *p* < 0.05. Effect sizes are reported as partial eta squared (*η*_p_^2^) where applicable.

## 4. Results

### 4.1. Group-Level Task-Switching Performance

Descriptive statistics (Table 1) summarize group-level performance in IES, RT, and ACC, along with standard deviations.

To establish whether short-form video exposure influenced overall task-switching performance, we first conducted one-way ANOVAs on switch trials collapsed across CTI conditions. Levene’s tests indicated homogeneity of variances for both reaction time (RT: *F*(2,69) = 0.781, *p* = 0.462) and accuracy (ACC: *F*(2,69) = 0.644, *p* = 0.528). No significant group difference was observed for RTs, *F*(2,69) = 1.249, *p* = 0.293. However, a marginal group difference emerged for accuracy rates, *F*(2,69) = 2.690, *p* = 0.075, with the short video group showing reduced accuracy relative to both the documentary (mean difference = −0.034, *p* = 0.041, LSD-corrected) and the no-video control group (mean difference = −0.032, *p* = 0.058, LSD-corrected).

Although these effects did not reach conventional significance thresholds, the trend suggests that brief exposure to short-form video content may be associated with diminished task-switching accuracy at a global level.

### 4.2. Condition-Specific Analysis: Preparatory Flexibility

To further examine how short-form video immersion influenced task preparation, a 3 (Group: short video vs. documentary vs. no-video control) × 2 (CTI: short vs. long) × 2 (Trial Type: repeat vs. switch) mixed-design ANOVA was conducted on IES, which provides an integrated measure of response speed and accuracy.

The results revealed a non-significant main effect of Group, and the assumption of sphericity was violated (Mauchly’s *W* = 0.757, *p* = 0.046). Greenhouse–Geisser correction was applied, yielding *F*(1.608, 36.993) = 2.494, *p* = 0.107, *η*_p_^2^ = 0.098.

Significant main effects were observed for both Trial Type, *F*(1, 23) = 139.233, *p* < 0.001, *η*_p_^2^ = 0.858, and CTI, *F*(1, 23) = 16.160, *p* = 0.001, *η*_p_^2^ = 0.413, indicating robust switch costs and a general performance benefit from extended preparation time.

A significant three-way interaction was detected (Group × Trial Type × CTI), with the sphericity assumption met (Mauchly’s *W* = 0.890, *p* = 0.279), *F*(2, 46) = 5.014, *p* = 0.011, *η*_p_^2^ = 0.179. Among the two-way interactions, a significant Group × CTI interaction emerged, *F*(2, 46) = 5.931, *p* = 0.005, *η*_p_^2^ = 0.205 (Mauchly’s *W* = 0.837, *p* = 0.141), suggesting that the benefit of extended preparation time differed across groups. In contrast, the Group × Trial Type interaction was non-significant, *F*(2, 46) = 0.938, *p* = 0.399, *η*_p_^2^ = 0.039 (Mauchly’s *W* = 0.964, *p* = 0.670), indicating that the magnitude of switch cost did not vary meaningfully between groups. The CTI × Trial Type interaction was significant, *F*(1, 23) = 7.927, *p* = 0.010, *η*_p_^2^ = 0.256, reflecting a general pattern whereby the advantage of longer preparation time was more pronounced on switch trials than on repeat trials.

**Figure 2:**
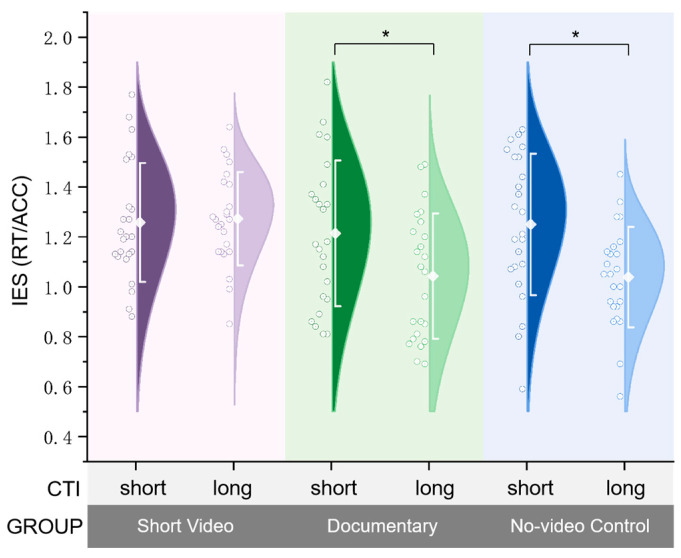
Task-switching performance (switch trials only), indexed by Inverse Efficiency Scores (IES) across CTI conditions for each group. Both the documentary and no-video control groups demonstrated a clear preparation benefit, with lower IES under long CTI compared to short CTI. In contrast, the short video group showed no difference in performance across CTI conditions, indicating a lack of proactive preparation. Error bars represent ±1 standard deviation. “*” represents *p* < 0.05.

To further unpack the significant three-way interaction (Group × Trial Type × CTI), we focused our simple-effects analyses on switch trials, where the effects of preparatory control are theoretically and empirically most pronounced (Figure 2). Switch trials require reconfiguration of task sets and engagement of top-down control, rendering them highly sensitive to preparatory manipulations such as CTI. In contrast, repeat trials, involving minimal cognitive updating, are relatively insensitive to preparation time and thus less diagnostic of proactive control. While repeat trials were included to validate the paradigm’s sensitivity to switch costs, our primary interest lies in the preparatory mechanisms reflected in switch performance.

In the short video group, CTI length had no significant effect on switch-trial IES, *t*(23) = −0.235, *p* > 0.05, mean difference = −0.015, indicating that extended preparation time did not enhance performance. By contrast, both the documentary and no-video control groups exhibited significant preparation benefits, with lower IES under long CTI compared to short CTI (Documentary: *t*(23) = 3.034, *p* = 0.039, mean difference = 0.172; Control: *t*(23) = 3.666, *p* = 0.020, mean difference = 0.211), suggesting preserved anticipatory control in these groups.

Taken together, these results demonstrate that even brief exposure to short-form video content selectively disrupts proactive preparation, abolishing the performance gains typically afforded by longer preparation time. While reactive control remains preserved, the goal-directed configuration of upcoming tasks appears impaired, reflecting a narrowed preparatory window and diminished capacity for anticipatory adjustment following digital media immersion.

The raw data supporting the findings of this study are available in the Supplementary Materials (File S1).

## 5. Discussion

In an era where short-form video platforms such as TikTok, Instagram Reels, and Kwai have become deeply embedded in daily life, understanding their cognitive implications is increasingly critical. The present study provides preliminary evidence that brief immersion in short-form video environments may transiently impair cue-based preparatory control—a specific anticipatory process that facilitates flexible task switching. Importantly, rather than indicating a generalized impairment in cognitive flexibility, the results isolate a specific disruption in anticipatory mechanisms—namely, the proactive use of task cues to configure internal goal states prior to switching. The result showed that participants exposed to short-form video content failed to benefit from extended CTIs, while those in the documentary and no-video groups demonstrated the expected reduction in switching costs under long CTIs.

Framed within the dual mechanisms of control framework (Braver, 2012), cognitive regulation involves both proactive control, which sustains task goals in anticipation of demands, and reactive control, which mobilizes responses in direct reaction to stimuli. Short-form video exposure, by reinforcing stimulus-driven engagement, may induce a reactive control bias, reducing reliance on proactive preparation. This strategic re-weighting likely impairs individuals’ ability to exploit predictive cues (such as those provided during long CTIs). In task-switching contexts, proactive control enables individuals to utilize preparatory intervals to reconfigure task sets and optimize performance (Meiran, 1996; Kiesel et al., 2010). The present results demonstrate that even brief interaction with fast-paced, fragmented digital content can transiently shift cognitive regulation toward a reactive mode, thereby reducing the efficiency of anticipatory preparation.

The timing-specific nature of the observed effects provides further evidence for a targeted disruption in proactive control mechanisms, rather than a global impairment. In our task design, the CTI was systematically manipulated to differentiate between reactive and proactive engagement. Short CTIs offer insufficient time for goal-directed preparation, thus primarily capturing reactive responding. Consistent with this logic, group differences were not observed under short CTIs, where proactive engagement is constrained and performance relies primarily on reactive control. It was only when ample time was available for anticipatory preparation that the short video group diverged—indicating a specific impairment in leveraging temporal affordances to reconfigure task sets. This selective deficit reflects a breakdown in strategic, goal-driven engagement, rather than a general decline in attention or motivation. This pattern aligns with dual-mechanism models (Braver, 2012), which propose that high-novelty environments—such as short-form video platforms—can temporarily suppress anticipatory regulation by biasing control toward stimulus-driven reactivity.

At a mechanistic level, short-form videos are purposefully engineered to maximize novelty, sensory stimulation, and emotional arousal—features that rapidly activate dopaminergic reward pathways and modulate prefrontal cortical activity (Ott & Nieder, 2019). Such overstimulation places considerable demands on working memory and attentional resources, potentially leaving insufficient capacity for maintaining internal task goals or preparing for upcoming cognitive demands. Moreover, repeated exposure to highly salient, algorithmically curated content may induce a state of cognitive “attentional saturation” (Firth et al., 2019; Coiera et al., 1996), in which individuals’ ability to sustain top-down, internally guided control becomes compromised. The diminished preparatory benefits observed in the short video group lend behavioral support to this theoretical account, suggesting that even brief exposure to hyper-stimulating digital content can transiently bias cognitive regulation away from strategic goal maintenance.

Beyond their theoretical value, the present findings bear substantial real-world implications. Cognitive flexibility is a foundational capacity that supports a wide range of adaptive outcomes, including academic achievement (Nakhostin-Khayyat et al., 2024), effective decision-making (Ionescu, 2012), and psychological resilience (Tong et al., 2024). That even brief and commonplace digital behaviors can transiently disrupt this function points to a subtle yet consequential cognitive cost of modern media consumption. This concern is particularly acute among adolescents and young adults, who not only represent the primary demographic for short-form video platforms but also possess heightened neural plasticity and increased vulnerability to environmental influences (Hadlington, 2015). Although the observed impairment may be short-lived, repeated disruptions to preparatory control may accumulate over time, potentially compounding into broader difficulties with learning, attention regulation, and adaptive functioning.

These findings highlight the urgent need for evidence-based interventions that move beyond simplistic metrics such as screen-time duration and instead target the structural characteristics of digital media that compromise cognitive regulation. Specifically, media literacy initiatives that cultivate attentional awareness, promote mindful and goal-directed technology use, and encourage paced, intentional engagement with information streams may offer promising avenues to buffer against the subtle but pervasive cognitive disruptions posed by fragmented digital ecosystems.

Nevertheless, several limitations warrant acknowledgment. First, although our task-switching paradigm offers a well-established behavioral index of cognitive flexibility, laboratory-based tasks may not fully capture the complexity and ecological demands of real-world cognitive functioning. Second, our sample comprised demographically homogeneous young adults with frequent exposure to short-form video content, which may constrain the generalizability of our findings to other populations—such as children, older adults, or individuals with atypical cognitive profiles—who may exhibit differential susceptibility to media-induced cognitive modulation. Third, we did not collect pre-exposure baseline measures of task-switching performance for each participant, limiting our ability to determine whether the observed group differences arose entirely from media exposure or may partially reflect pre-existing individual differences in executive control. Although random assignment and the inclusion of a no-video control group help mitigate this concern, they cannot fully replace within-subject comparisons. Future studies employing repeated-measures or crossover designs will be critical for isolating causal effects with greater precision. Finally, individual difference factors—such as baseline attentional capacity, habitual media use patterns, or developmental stage—likely modulate the impact of digital immersion on cognitive flexibility and should be explicitly modeled in subsequent investigations. Longitudinal and neurophysiological approaches will also be essential to delineate the temporal dynamics and underlying mechanisms of these effects.

## 6. Conclusions

This study identifies a specific, CTI-dependent impairment in proactive control following brief exposure to short-form video content. While performance under short CTI remained unaffected, participants in the short video group failed to benefit from longer intervals that typically support anticipatory preparation—unlike those in the documentary and control groups. These findings isolate a transient, media-induced disruption in cue-based task-set reconfiguration, a core subcomponent of cognitive flexibility. By linking immersive digital environments to failures in anticipatory regulation, this research highlights a previously underexplored short-term vulnerability within executive function systems. As short-form video platforms continue to proliferate and permeate daily life, especially among younger populations, understanding their subtle yet systematic cognitive consequences represents a pressing challenge for interdisciplinary research. Addressing this challenge will require not only refined theoretical models of media–cognition interaction but also evidence-based strategies to support attentional regulation and adaptive functioning in increasingly fragmented digital ecosystems.

## Figures and Tables

**Table 1:** Mean IES, RT, and ACC for each group, with standard deviations.

Group	Mean IES (SD)	Mean RT (SD) (ms)	Mean ACC (SD)
Short Video	1.139 (0.244)	1.108 (0.179)	0.876 (0.062)
Documentary	1.027 (0.272)	1.020 (0.217)	0.910 (0.057)
No-Video Control	1.041 (0.249)	1.048 (0.191)	0.908 (0.052)

## Data Availability

The data will be made available to interested scientists, upon requests addressed to the authors, following article acceptance and IRB approval for data sharing.

## Supplementary Materials

The following supporting information can be downloaded at: https://www.mdpi.com/article/10.3390/bs15081070/s1, File S1: Trial-level raw data for each participant, including condition labels, stimulus properties, response times, and accuracy (Excel format). Suitable for replication or reanalysis.

## Abbreviations

The following abbreviations are used in this manuscript:

RT	Reaction time
ACC	Proportion of correct responses
CTI	Cue–target interval
IES	Inverse efficiency score
